# Effect of Peripheral Magnetic Stimulation for Dysphagia Rehabilitation: A Systematic Review

**DOI:** 10.3390/nu14173514

**Published:** 2022-08-26

**Authors:** Na-Kyoung Hwang, Ji-Su Park, Jong-Bae Choi, Young-Jin Jung

**Affiliations:** 1 Department of Occupational Therapy, Seoul North Municipal Hospital, Seoul 02062, Korea; 2 Research Institute for Korean Medicine, Pusan National University, Yangsan 50612, Korea; 3 Department of Occupational Therapy, Sangji University, Wonju 26339, Korea; 4 School of Healthcare and Biomedical Engineering, Chonnam National University, Yeosu 59626, Korea

**Keywords:** repetitive peripheral magnetic stimulation, dysphagia, suprahyoid muscles, systematic review

## Abstract

Recently, a therapeutic method to stimulate the suprahyoid muscle using peripheral magnetic stimulation for dysphagia rehabilitation has been reported. However, clinical evidence, application protocol, and intervention method remain unclear. Therefore, a systematic review of the published literature is needed. The objective of this study was to systematically review clinical studies of peripheral magnetic stimulation applied for rehabilitation of dysphagia. Issues to be considered in future studies are also suggested. This systematic review performed a literature search of four databases (Medline, Embase, CINAHL, and Web of Science) to identify relevant studies published on the application of repetitive peripheral magnetic stimulation (rPMS) for swallowing-related muscles between 2010 and 2022. Seven studies were reviewed. Randomized controlled trials and one-group pre–post, case study designs were included. In the included studies, rPMS was applied to strengthen the submental suprahyoid muscles. The intervention regime varied. The rPMS was applied at a frequency of 30 Hz for 2 s. Rest time ranged from 8 s to 27–28 s. The number of intervention sessions ranged from 2–3 to 30. The intensity ranged from pain-inducing minimum intensity (90% of maximum stimulus output) to non-painful intensity (70–80% of maximum intensity). The rPMS on the suprahyoid muscles had positive effects on physiological changes in the swallowing function, such as displacement of the hyoid bone, muscle strength (cervical flexor, jaw-opening force), swallowing safety, swallowing performance, and swallowing-related quality of life. Participants also reported little pain and adverse reactions during rPMS. Although rPMS is a therapeutic option that can help improve the swallowing function as a non-invasive stimulation method in the rehabilitation of dysphagia, clinical evidence is needed for the development of clear stimulation protocols and guidelines.

## 1. Introduction

Anterior and superior displacement of the hyoid bone or “excursion” is the most common indicator of the physiological initiation of swallowing. It occurs when the bolus enters the pharynx. The suprahyoid muscles pull the hyoid bone anterior and upward along with contraction of the tongue base to close the epiglottis and open the upper esophageal sphincter (UES), thereby protecting the airway and contributing to the safe and efficient movement of bolus into the esophagus. Therefore, the suprahyoid muscles play an important role in the normal swallowing mechanism [1,2]. The suprahyoid muscles are a muscle group consisting of four muscles located in front of the neck: Geniohyoid, mylohyoid, digastric anterior and posterior belly, and stylohyoid muscles [3].

The effectiveness of neuromuscular electrical stimulation (NMES) has been demonstrated in dysphagic patients with reduced hyoid elevation [4,5]. Surface electrodes can be applied to the suprahyoid muscles to directly stimulate these muscles that are the cause of dysphagia. In addition, NMES can be applied to the infrahyoid muscles that depress the hyoid. It has been used as a resistance training to strengthen the suprahyoid muscles [6,7,8]. However, it is a very challenging work to elevate the hyoid through continuous swallowing against the depressed hyoid [6]. It might be difficult to apply NMES to patients with seriously reduced hyoid elevation. Regardless of the muscle area being applied, it is impossible to apply NMES to achieve strong stimulation stimulus since high-intensity stimulation, known as the exercise level, can cause pain and make the patient feel uncomfortable, which may eventually cause difficulties in treatment compliance. Moreover, electrical stimulation using surface electrodes has the potential to stimulate areas other than the target muscle [9,10,11].

Repetitive peripheral magnetic stimulation (rPMS) is a non-invasive treatment modality developed for therapeutic neuromodulation of movement disorders [12]. Magnetic stimulation uses a time-varying electromagnetic field to induce an eddy current in an adjacent volume without passing through the skin, thereby activating the nerve muscle without stimulating skin nociceptors [13]. Stimulation coils (magnetic field generators) are placed over predominantly paralyzed muscles of arms, legs or torso. Both NMES and rPMS are mainly applied to people with motor dysfunction as a result of damage to the brain or nerves. However, rPMS can provide painless stimulation to deep muscles that NMES cannot reach [14]. In addition, unlike NMES, which recruits cutaneous receptors that generate noisy signals, rPMS can generate proprioceptive information during muscle contraction. It affects brain plasticity through proprioceptive feedback and improves the sensorimotor system [15]. In general, the rPMS device is not suitable for small muscles since it is bulky and difficult to provide controlled local stimulation [15]. However, recently, a device with a small coil for stimulating the suprahyoid muscle has been developed and applied. It has the potential to replace NMES, which has been considered as a therapeutic stimulation modality to improve reduced hyoid elevation [16].

Clinical trials have been conducted to demonstrate the effectiveness of rPMS for people with functional disabilities [17,18]. Recently, Sakai et al. [19] have performed a meta-analysis of the effectiveness and safety of rPMS on the functional ability and daily living activities in stroke patients. However, in these studies, rPMS has been mainly applied to the upper and lower limbs with reduced function or paralysis. Although studies on the effectiveness of PMS in swallowing disorders have been conducted in recent years, no reviews have been conducted. It is unclear whether rPMS is useful for improving suprahyoid muscles’ function in patients with dysphagia and what regime (frequency, stimulation session, sessions in treatment, and intensity) should be performed. Therefore, the purpose of this review was to summarize PMS regimes and their clinical effectiveness in dysphagia reported to date.

## 2. Materials and Methods

### 2.1. Search Strategy and Selection Criteria

**Figure 1:**
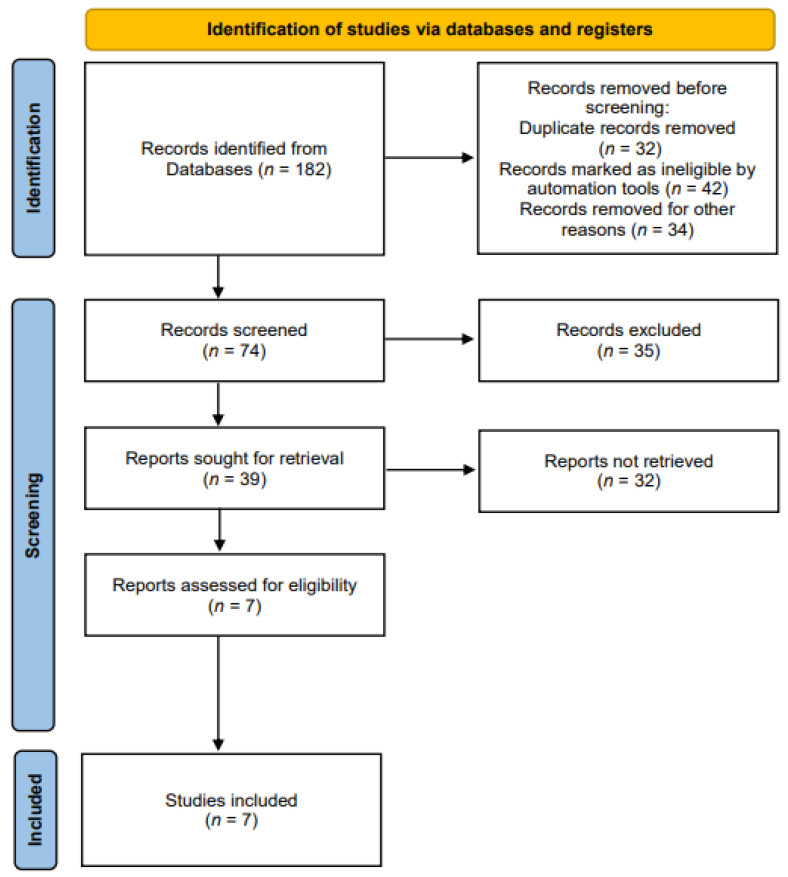
The preferred reporting items for systematic reviews and meta-analyses (PRISMA) flow chart.

Related studies were identified according to the Preferred Reporting Items for Systematic Reviews and Meta-Analyses (PRISMA) guidelines [20] (Figure 1). The literature search was performed according to the PICO search strategy [21]: P (Population), normal adults or patients with dysphagia; I (Intervention), rPMS for strengthening swallowing-related muscles; C (Comparison), no control limit; O (Outcome), measurement of swallowing-related functions, structural changes, and myophysiological changes.

We selected the four databases (Medline, Embase, CINAHL, and Web of Science) to identify the relevant studies published on rPMS interventions for strengthening swallowing-related muscles between 2010 and 2022. We used the following keywords: (magnetic*) AND (stimu* OR treatment OR rehabilit*) AND (dysphagia OR deglutition disorder OR swallowing disorder). Search terms were selected to maximize both search sensitivity and specificity. The selection process was conducted in two phases. In phase one, two reviewers (N.K. Hwang and J.S. Park) independently screened the titles and abstracts of all searched materials. Articles that did not meet the eligibility criteria were excluded. In phase two, the same reviewers independently screened full texts of studies. Those that failed to meet the study eligibility criteria were excluded.

Eligibility criteria were: (1) Studies applying rPMS to swallowing-related muscles, (2) studies with outcomes focusing on swallowing-related functions, structural changes, and myophysiological changes, (3) studies on healthy adults or patients with dysphagia. We excluded the following: (1) Non-English publications, (2) animal studies, (3) expert opinion articles, (4) studies that provided insufficient information to extract data, (5) studies that failed to report dose outcomes. Study titles and abstracts were examined after the initial search. Then, full texts of eligible studies were obtained. Manuscripts were searched for the presence of inclusion and exclusion criteria. A consensus to include each study in the systematic review was reached between the authors.

### 2.2. Quality Appraisal

**Figure 2:**
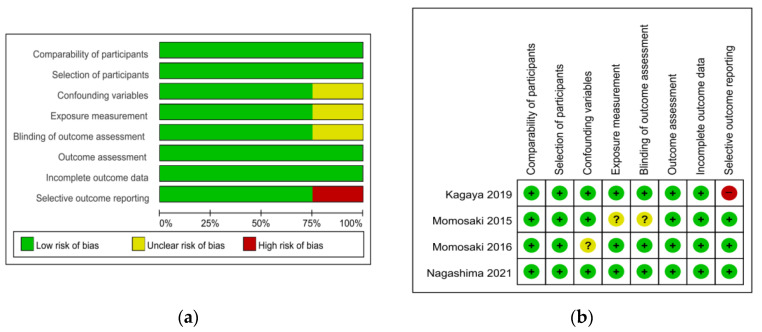
Quality assessment of the included studies using ROBANS. (**a**) ROBANS graph and (**b**) ROBANS summary; +: Low risk of bias; ?: Unclear risk of bias; −: High risk of bias; ROBANS: Risk of bias assessment tool for non-randomized studies.

The risk of bias tool for randomized trials (RoB 2.0) was used to appraise two randomized controlled trial (RCT) studies. For the two RCT studies, the overall bias was ‘low risk’ for one [22] and ‘some concern’ for the other [23]. Items of the randomization process and the measurement of outcome showed ‘some concern’ of bias (Table 1). The Risk of Bias Assessment Tool for Non-Randomized Studies (RoBANS) was used to appraise the quality of four non-randomized trials except for one case study [24], which was a non-comparative study. Most of the items were rated as ‘low risk’. Confounding variables [25], exposure measurement, and blinding of outcome assessment [26] were rated as ‘unclear’ and selective outcome reporting [27] was rated as ‘high risk’ (Figure 2). 

## 3. Results

### 3.1. Participants’ Characteristics

We included seven studies with a total of 96 participants (sample size range: *n* = 2–24). Participants were healthy adults (*n* = 4), poststroke dysphagic patients (*n* = 2), dysphagic patients with reduced hyoid elevation including disuse syndrome after aspiration pneumonia and dermatomyositis (*n* = 1).

### 3.2. Intervention Approaches

Active rPMS was applied directly to the submental suprahyoid muscle. One [28] of the seven studies performed the EMG-triggered rPMS. The included studies used varied protocols of rPMS. Regarding the frequency of rPMS, all studies applied 30 Hz for 2 s. One study [25] compared the outcomes of 30 Hz for 2 s and 20 Hz for 3 s. Rest time (stimulation off) ranged from 8 s [22] to 27–28 s [23,25,26]. While four studies conducted outcome measurements during on and off stimulation [27,28] or performed immediate measurements on the day of the intervention [23,25], multiple sessions of rPMS were conducted with an intervention period of 6 days or longer in three studies [22,24,26]. The number of intervention sessions ranged from 2–3 [24] to 30 [22]. The intensity ranged from pain-inducing minimum intensity (90% of maximum stimulus output) [23,25,26] to non-painful intensity (70–80% of maximum intensity) [22,28]. Co-exercises were performed as conventional dysphagia rehabilitation (oral stretching, tongue push-up exercise, and isokinetic HLE) after stimulation in two studies [24,26]. Two studies included a group for comparison [22,23], with the comparison group receiving a head-lift exercise (HLE) or sham stimulation. Sham stimulation was applied in a way that the coil was turned on but the target site was not stimulated [23]. Lengths of interventions ranged from 1 to 6 weeks in three studies [22,24,26]. Four studies [23,25,27,28] conducted immediate outcome measurement pre–post interventions on a single day (Table 2).

### 3.3. Outcome Measures

Table 2 and Table 3 show the outcomes of rPMS interventions.

#### 3.3.1. Physiological Changes in Swallowing Function

Displacement hyoid bone (*n* = 4), opening width of UES (*n* = 2), inter-swallow interval (ISI) (*n* = 1), laryngeal elevation delay time (LEDT) (*n* = 1), swallowing speed and capacity (*n* = 1), muscle strength, cervical flexor, jaw-opening force (*n* = 2), and UES relaxation time (*n* = 1) were found to be outcomes of physiological changes in the swallowing function. In the displacement hyoid bone, only one study showed a significant improvement in the anterior movement of the hyoid bone in the pre–post comparison [28]. Moreover, positive effects were reported for outcomes of the opening width of UES [28], LEDT [26], swallowing speed and capacity [23], and muscle strength [22].

#### 3.3.2. Swallowing Safety

The penetration aspiration scale (PAS) to quantify the swallowing safety was measured in one study [26]. It reported a significant improvement in PAS before and after stimulation (*p* < 0.01).

#### 3.3.3. Swallowing Performance

Regarding the functional oral intake scale (FOIS) to document the functional level of the oral intake of food and liquid, Mann assessment of swallowing ability (MASA) to comprehensively evaluate participants’ swallowing ability was used in one study [26]. It showed the significant results only in MASA.

#### 3.3.4. Quality of Life

Swallowing quality of life (SWAL-QOL) was used to assess the psychosocial aspects of patients with swallowing disorders in one study [26]. It showed a significant improvement in SWAL-QOL after sPMS intervention.

#### 3.3.5. Swallowing Biomechanics

Surface electromyography (sEMG)-median frequency (MF) rate (*n* = 2), tongue pressure (*n* = 2), maximum pre-opening, post-closure, nadir UES pressure, and velopharyngeal pressure (*n* = 1) were measured as swallowing biomechanics outcomes. Regarding the sEMG-MF outcome, when muscle fatigue increased, it decreased to a negative value. When muscle fatigue decreased, it approached zero. In one study [22], there was no significant change in suprahyoid, infrahyoid or sternocleidomastoid muscles in either group between pre-test and post-test. There was also no significant difference between the groups [22]. However, in another study [24], the MF rate of suprahyoid muscles showed a tendency of improvement between pre-test and post-test. Regarding the UES pressure and velopharyngeal pressure outcomes, only the maximum post-closure UES pressure showed a significant improvement between pre-test and post-test [28].

**Table 2:** Summary of studies investigating the use of rPMS.

Author (Year)	Design Participants	Intervention Regime	Outcome Measure Assessment	Key Finding
Ogawa et al. 2020 [22]	RCT Healthy adults rPMS = 12 HLE = 12	Active rPMS	Cervical flexor strength: dynamometer JOF: JOF measurement device Tongue pressure: tongue pressure measurement device Muscle fatigue of the hyoid and laryngeal muscles: sEMG; MF rate Displacement of the hyoid bone, opening width of UES during 10 mL of liquid swallow: VFSS + image J program Training performance rate Pain: NRS	Significantly increased cervical flexor strength in IG at pre–post test (*p* = 0.009); no significant differences between the groups Significantly improved tongue pressure in both groups at pre–post test (*p* = 0.006, 0.006, respectively); no significant differences between the groups Improving trend of JOF in both groups at pre-post test; no significant change in both groups No significant change in the MF rate of the anterior belly of the digastric sternohyoid, sternocleidomastoid muscle in both groups at pre–post test; no significant differences between the groups No significant change in anterior and superior hyoid bone displacement, and UES opening width in both groups at pre–post test; no significant differences between the groups No significant differences in the training performance rate between the two groups (no dropouts) No significant differences in NRS between the groups after the third set of training
		Frequency: 30 Hz Stimulation session: 2 s on and 8 s off Number of sessions in treatment: 30 (3 times a day, 5 days a week, 2 weeks) Intensity: 70% of maximal stimulator output		
		HLE		
		Isometric HLE for 1 min (3 times a day, 5 days a week, 2 weeks) Isotonic HLE for 30 consecutive repetitions (3 times a day, 5 days a week, 2 weeks)		
Momosaki et al., 2014 [23]	RCT Poststroke dysphagic patients (IG = 10, CG = 10)	Active rPMS	Swallowing ability: timed water swallow test, ISI, swallowing volume velocity (speed), volume per swallow (capacity)	Significant improvement in speed and capacity of swallowing after stimulation in IG compared with CG (*p* = 0.008, 0.005, respectively) No significant difference in the ISI between the groups No adverse reactions throughout the stimulation Subjective reporting in IG: feelings of diminished anxiety about choking during water swallowing and easier production of the swallowing reflex after PMS
		Frequency: 30 Hz Stimulation session: 2 s on and 28 s off Number of sessions in treatment: 1 session on a single day Duration of stimulation: repetition for 10 min Intensity: 90% of the minimal intensity causing pain		
		Sham rPMS		
		Frequency: n/a Duration of stimulation: 10 min Intensity: 0% (using non-active coil; at the same site as the active rPMS group)		
Mori et al., 2019 [24]	Case study Dysphagic patients with reduced hyoid elevation *n* = 2	Active rPMS	Displacement of the hyoid bone: VFSS + image measurement software program Cervical flexor strength: dynamometer JOF: JOF measurement device Muscle fatigue of suprahyoid and infrahyoid muscles: sEMG; MF rate Pain: NRS	Case 1
				- Improved anterior and upward hyoid movement during 4 mL of 1% nectar-thick swallow (8.0, 9.0 mm increase in pre–post test, respectively) - Improved cervical flexor, jaw-opening muscle strength (3.5, 0.7 kgf increase in pre–post test, respectively) - No pain reported: NRS = 0 - No complications reported
		Frequency: 30 Hz Stimulation session: 2 s Number of sessions in treatment: 2–3 (1 time a day, at least 5 days a week, 6 weeks) Intensity: level enough to generate hyoid bone movement without causing pain Co-exercise: conventional dysphagia rehabilitation		Case 2
				- Improved jaw-opening muscle strength: 1.7 kgf at pre-test, 7.5 kgf at post-test - Declined fatigue in suprahyoid muscles: MF rates −3.03 at pre-test, −1.45 at post-test - Increased duration in neck flexion retention in the supine position: 10 s at pre-test, 30 s at post-test - No pain reported (NRS = 0) - No complications reported - Subjective reporting: alleviated neck stiffness and reduced fatigue upon eating after PMS
Momosaki et al., 2016 [25]	One group pre–post Healthy adults *n* = 10	Active rPMS	Swallowing biomechanics: MEP	Significantly increased MEP in both 20 and 30 Hz before, immediately after, and 30 min after (*p* < 0.05), with the increase maintained until 30 min after stimulation (*p* < 0.05) No significant difference in MEP immediately after stimulation between the 20 and 30 Hz frequencies No adverse reactions throughout the stimulation
		Frequency: 20/30 Hz Stimulation session: 20 Hz for 3 s on and 27 s off/30 Hz for 2 s on and 28 s off Number of sessions in treatment: 20 (1 time each Hz on different days) Duration of stimulation: 10 min Intensity: 90% of the minimal intensity level causing pain		
Momosaki et al., 2015 [26]	One group pre–post Poststroke dysphagic patients (*n* = 8)	Active rPMS	Swallowing function: VFSS; LEDT Swallowing safety: VFSS; PAS Swallowing performance: FOIS; MASA QOL: SWAL-QOL	Significant improvement in PAS, LEDT, MASA, and SWAL-QOL (*p* = 0.01. 0.02, 0.01, 0.01, respectively) No significant effect on the FOIS (*p* = 0.08) No adverse reactions throughout the stimulation
		Frequency: 30 Hz Stimulation session: 2 s on and 27 s off Number of sessions in treatment: 20 (2 times a day, 6 consecutive days) Duration of stimulation: 10 min Intensity: 90% of the minimal intensity causing pain Co-exercise: conventional dysphagia rehabilitation (oral stretching, tongue push-up exercise, and isokinetic HLE) over 20 min		
Kagaya et al., 2019 [27]	One group pre–post Healthy adults *n* = 12	Active rPMS	Displacement of the hyoid bone: VFSS + image measurement software program Pain: NRS	Degree of hyoid bone movement during stimulation: anterior 10.9 ± 2.8 mm and superior 8.3 ± 4.1 mm; similar degree to normal drinking (anterior: 12.9 ± 3.4, superior: 6.5 ± 3.4) Reported pain level: NRS = 1
		Frequency: 30 Hz Stimulation session: 2 s Number of sessions in treatment: one session on a single day (on and off stimulation) Intensity: level enough to generate hyoid bone movement without causing intolerable pain		
Nagashima et al., 2021 [28]	One group pre–post Healthy adults (*n* = 20)	EMG-triggered rPMS	Displacement of the hyoid bone, opening width of UES: VFSS + image measurement software program Pressure topography: manometry	Significantly extended the movement time of the hyoid bone with magnetic stimulation during saliva and liquid swallow (*p* < 0.001) Significant increase in the forward maximum movement distance of the hyoid bone with magnetic stimulation during liquid swallow (*p* < 0.05) No significant difference in the upward maximum movement distance between magnetic stimulation and non-magnetic stimulation during saliva and liquid swallow Significant increase in the opening width of the UES, and forward hyoid displacement at the maximum UES opening with magnetic stimulation during liquid swallow (*p* < 0.01) Significant decrease in the maximum post-closure UES pressure with magnetic stimulation during saliva and liquid swallow (*p* < 0.05); no significant difference in maximum velopharyngeal, tongue-base, pre-opening UES pressure, nadir UES pressure, and UES relaxation time
		Frequency: 30 Hz Stimulation session: 2 s Number of sessions in treatment: one session on a single day (on and off stimulation) Intensity: 70–80% of maximal stimulator output (the intensity that does not cause pain)		

RCT: Randomized controlled trial; IG: Intervention group; CG: Control group; rPMS: Repetitive peripheral magnetic stimulation; HLE: Head lift exercise; UES: Upper esophageal sphincter; MF: Median frequency; sEMG: Surface electromyography; VFSS: Video fluoroscopic swallowing study; JOF: Jaw-opening force; NRS: Numerical rating scale; PAS: Penetration aspiration scale; LEDT: Laryngeal elevation delay time; FOIS: Functional oral intake scale; QOL: Quality of life; SWAL-QOL: Swallowing quality of life; MASA: Mann assessment of swallowing ability; ISI: Inter-swallow interval; MEP: Motor-evoked potential; TMS: Transcranial magnetic stimulation.

**Table 3:** Summary of selected outcomes.

Outcome		Number of Studies That Assessed This Outcome	Study	Effect
Physiological changes in swallowing function	Displacement in the hyoid bone	4	Ogawa, 2020 [22]	– (forward, upward)
			Kagaya, 2019 [27]	x
			Mori, 2019 [24]	^ (forward, upward)
			Nagashima, 2021 [28]	+++ (forward), – (upward)
	Opening width of UES	2	Ogawa, 2020 [22]	–
			Nagashima, 2021 [28]	+++
	LEDT	1	Momosaki, 2015 [26]	+++
	ISI	1	Momosaki, 2014 [23]	^
	Swallowing speed	1	Momosaki, 2014 [23]	+++
	Swallowing capacity	1	Momosaki, 2014 [23]	+++
	Muscle strength	2	Ogawa, 2020 [22]	++ (cervical flexor), ^(JOF)
			Mori, 2019 [24]	^ (cervical flexor, JOF)
	UES relaxation time	1	Nagashima, 2021 [28]	–
Swallowing safety	PAS	1	Momosaki, 2015 [26]	+++
Swallowing performance	MASA	2	Momosaki, 2015 [26]	+++
	FOIS		Momosaki, 2015 [26]	^
Quality of life	SWAL-QOL	1	Momosaki, 2015 [26]	+++
Swallowing biomechanics	EMG-MF rate	2	Ogawa, 2020 [22]	–
			Mori, 2019 [24]	^
	Tongue pressure	2	Ogawa, 2020 [22]	+
			Nagashima, 2021 [28]	–
	Maximum post-closure UES pressure	1	Nagashima, 2021 [28]	+++
	Maximum velopharyngeal pressure	1	Nagashima, 2021 [28]	–
	Maximum pre-opening UES pressure	1	Nagashima, 2021 [28]	–
	Maximum nadir UES pressure	1	Nagashima, 2021 [28]	–
Neurophysiological changes	MEP	1	Momosaki, 2016 [25]	+++
Other measures	Pain	3	Ogawa, 2020 [22]	NRS = 0
			Kagaya, 2019 [27]	NRS (median) = 1
			Mori, 2019 [24]	NRS = 0
	Compliance	1	Ogawa, 2020 [22]	#
	Adverse reactions	4	Mori, 2019 [24]	*
			Momosaki, 2016 [25]	*
			Momosaki, 2015 [26]	*
			Momosaki, 2014 [23]	*

+++: Statistically significant effect; ++: Greater improvement in intervention group than control but between group difference not significant; +: Significant improvement in both groups but between group difference not reported or not significant; –: No reported change between the groups; x: Effect-related data not shown; ^: Within-group improvement not significant; #: High compliance data from the number of participants; *: No adverse reactions as reported by the participants; UES: Upper esophageal sphincter; ISI: Inter-swallow interval; LEDT: Laryngeal elevation delay time; FOIS: Functional oral intake scale; SWAL-QOL: Swallowing quality of life; MASA: Mann assessment of swallowing; EMG: Electromyography; MF: Median frequency; MEP: Motor-evoked potential; JOF: Jaw-opening force; NRS: Numerical rating scale.

#### 3.3.6. Neurophysiological Changes

To identify the cortical excitability of muscles related to swallowing, the motor-evoked potential (MEP) amplitude of suprahyoid muscles was analyzed in one study [25]. It compared MEP at 30 and 20 Hz, both Hz showed significant improvement in MEP immediately after stimulation (*p* < 0.05). In addition, the MEP was maintained until 30 min after stimulation (*p* < 0.05).

#### 3.3.7. Other Measures: Pain, Compliance, and Adverse Reactions

The numeric rating scale (NRS) was used to assess pain intensity during rPMS in three studies [22,24,27]. They reported that participants’ average NRS scores were 1 or 0. One study assessed the training performance rate to compare compliance with rPMS and HLE [22], reporting no significant difference between the two groups. Adverse reactions were collected in four studies [23,24,25,26]. There were no adverse reactions to rPMS reported by the participants in those studies.

## 4. Discussion

Magnetic stimulation is a technique that can stimulate not only central nerves, but also peripheral nerves. It is applied in various rehabilitation areas, such as cranial nerve stimulation, musculoskeletal rehabilitation, and pain control. In particular, many attempts have been made to stimulate the cerebral cortex related to swallowing to improve the swallowing function through central nerve stimulation in dysphagia rehabilitation. Numerous previous studies have reported its mechanism and clinical effect [29,30,31,32,33].

Recently, several investigators have reported the effectiveness of peripheral nerve stimulation using the magnetic stimulation technique for dysphagia rehabilitation [22,23,24,25,26,27,28]. The reason for applying magnetic stimulation to the rehabilitation of dysphagia is due to the fact that it has better penetration into soft tissues, such as muscles, than the electrical stimulation used in the past. In addition, it does not have direct contact with the skin, thus causing no pain. Electrical stimulation, which is commonly used in clinical practice, requires a fairly high intensity to contract the swallowing muscle through skin penetration, which is called motor level [34]. Since muscle contraction through motor level causes discomfort or pain, it is disadvantageous in terms of compliance with rehabilitation. In addition, since it is a skin-to-skin contact method using a surface electrode, it is easily exposed to problems, such as skin redness and troubles.

To date, there have been seven studies applying PMS to the rehabilitation of dysphagia. Although there were differences in research methodologies, such as subject characteristics, intervention methods, and evaluation, all of them stimulated the suprahyoid muscles in the pharyngeal phase as a target [22,23,24,25,26,27,28]. The effect of stimulation is effective in increasing cervical flexor strength, improving hyoid bone movement, increasing UES opening, increasing tongue pressure [22,27,28], as well as reducing airway aspiration and improving the swallowing function [26] as a result of immediate or interventional results. Side effects and dropouts due to PMS were not reported in any studies.

The effect of PMS on dysphagia can be divided into two mechanisms: PNS and CNS. It can be explained by several reasons as follows. First, PMS might have a positive effect on the major functions related to swallowing through myophysiologic changes, such as muscle activity, increase in muscle strength, and increase in muscle volume. PMS can cause immediate contraction of the suprahyoid muscles, which can directly induce an increase in the movement of the hyoid bone. In addition, stimulation of the suprahyoid muscles using PMS can increase the number of motor units and cause an increased discharge rate of motor units [35,36]. Moreover, it can induce sufficient movement of the hyoid bone during swallowing, which affects the decrease in aspiration through airway protection and the increase in the opening of the UES [37,38]. Furthermore, PMS applied to the anterior neck can induce muscle strength increase through tongue muscle stimulation, which has a positive effect on tongue pressure increase during swallowing [22]. This helps reduce the airway aspiration and clearance of vallecular residues from a functional aspect of swallowing through the formation of high negative pressure during swallowing. In the anatomical structure, the tongue and suprahyoid muscles are partially interdigitated based on the hyoid bone. The genioglossus is a large muscle. Its fibers can interdigitate with those of the geniohyoid muscle [39,40]. Therefore, PMS in the anterior cervical region can stimulate not only the suprahyoid muscles, but also the tongue muscle.

Second, PMS can induce swallowing-related CNS changes through afferent stimulation. One of the afferent pathways of the swallowing reflex is the sensory branch of the vagus nerve, which arises from the pharyngeal mucosa. Stimulation of the vagus nerve could lead to the excitation of afferent input from the oropharynx and subsequently act on the swallowing reflex center in the medulla oblongata and on the cerebral cortex, causing neuromodulation and excitation of the swallowing response [23,41]. Therefore, PMS can contribute to the improvement in the swallowing function through stimulation of swallowing cortical activity or corticobulbar tracts, as well as stimulation of suprahyoid muscle [26]. PMS of the oropharyngeal or laryngeal region can also facilitate cortical neuronal activity in the swallowing area and the swallowing central pattern generator. A previous study has reported that stimulation of lower motor neurons can induce changes in the cerebral cortex, particularly inducing muscle contraction and corticospinal plasticity [42]. PMS can induce proprioceptive inflow that influences motor planning at the cortical level [42]. Therefore, stimulation of mandibular nerves by PMS can increase excitability of the motor cortex related to swallowing.

PMS was found to be safe and effective as a non-invasive therapeutic stimulation method that could ultimately help improve the swallowing function through changes in the CNS and PNS in the rehabilitation of dysphagia. However, there are issues to be considered when applying PMS to patients with dysphagia. First, since the swallowing muscle is a very small muscle located in a narrow space under the hyoid bone and chin, accurate stimulation focusing is important. However, the PMS equipment currently used is not a device developed for stimulating the swallowing muscle. Therefore, it is not a dedicated device considering the anatomical structure and characteristics of the swallowing muscle. Moreover, the protocol for optimal stimulation is not yet clear. As a result, clearer evidence is needed through coil design, which reflects the structure and characteristics of the swallowing muscle and various stimulation protocols for the effective rehabilitation of dysphagia.

## 5. Conclusions

This review identified studies using PMS for the rehabilitation of dysphagia. PMS is a safe, non-invasive stimulation method that can be used as a therapeutic method to help restore various functions related to swallowing through suprahyoid muscle stimulation.

## Figures and Tables

**Table 1:** Risk of bias summary in RCT literatures.

Author Year	Ogawa 2019 [22]	Momosaki 2014 [23]
Randomization process	some concern	some concern
Deviations from intended interventions	low risk	low risk
Missing outcome data	low risk	low risk
Measurement of the outcome	low risk	some concern
Selection of the reported result	low risk	low risk
Overall bias	low risk	some concern

RCT: Randomized controlled trial; RoB: Risk of bias.

## Data Availability

The data that support the findings of this study are available from the corresponding author, upon reasonable request.
